# Breast cancer in East Africa: Prevalence and spectrum of germline SNV/indel and CNVs in *BRCA1* and *BRCA2* genes among breast cancer patients in Tanzania

**DOI:** 10.1002/cam4.5091

**Published:** 2022-07-31

**Authors:** Linus P. Rweyemamu, Büşra K. Gültaşlar, Gokce Akan, Nazima Dharsee, Lucy A. Namkinga, Sylvester L. Lyantagaye, Hülya Yazıcı, Fatmahan Atalar

**Affiliations:** ^1^ Department of Molecular Biology and Biotechnology University of Dar es Salaam Dar es Salaam Tanzania; ^2^ Mbeya College of Health and Allied Sciences University of Dar es Salaam Mbeya Tanzania; ^3^ Division of Cancer Genetics, Department of Basic Oncology, Institute of Oncology Istanbul University Istanbul Turkiye; ^4^ DESAM Research Institute Near East University Nicosia Cyprus; ^5^ MUHAS Genetic Laboratory, Department of Biochemistry Muhimbili University of Health and Allied Sciences Dar es Salaam Tanzania; ^6^ Academic, Research and Consultancy Unit Ocean Road Cancer Institute Dar es Salaam Tanzania; ^7^ Department of Medical Biology and Genetics, Faculty of Medicine Istanbul Arel University Istanbul Turkiye; ^8^ Department of Rare Diseases, Child Health Institute Istanbul University Istanbul Turkiye

**Keywords:** *BRCA1/2*, breast cancer, germline mutations, next‐generation sequencing, Tanzania

## Abstract

**Background:**

Growing prevalence and aggressiveness of breast cancer (BC) among East African women strongly indicate that the genetic risk factor implicated in the etiology of the disease may have a key role. Germline pathogenic variants in *BRCA1* and *BRCA2* (*BRCA1/2*) are known to increase the lifetime risk of BC. This study investigated the prevalence and spectrum of germline single nucleotide variant/insertion and deletion (SNV/indel), and copy number variations (CNVs) in *BRCA1/2* among Tanzanian BC patients, and evaluated the associations of identified variants with patient's socio‐demographic and histopathological characteristics.

**Methods:**

One hundred BC patients were examined for *BRCA1/2* variants using next‐generation sequencing (NGS). Sanger sequencing and multiplex ligation‐dependent probe amplification (MLPA) assay were performed for the confirmation of SNV/indel and CNVs, respectively.

**Results:**

Six germline SNV/indel pathogenic variants were detected from six unrelated patients. Five of these variants were identified in *BRCA1*, and one in *BRCA2*. We also identified, in one patient, one variant of uncertain clinical significance (VUS). CNV was not detected in any of the BC patients. Furthermore, we found that in our cohort, *BRCA1/2* variant carriers were triple‐negative BC patients (*p =* 0.019).

**Conclusions:**

Our study provides first insight into BC genetic landscape by the use of NGS in the under‐represented East African Tanzanian populations. Our findings support the importance of genetic risk factors in BC etiology in Tanzania and showed a relatively high overall prevalence (6%) of germline *BRCA1/2* pathogenic variants in BC patients. Therefore, our results indicate that *BRCA1/2* pathogenic variants may well contribute to BC incidence in Tanzania. Thus, the identification of frequent variants in *BRCA1/2* genes will enable implementation of rapid, inexpensive population‐specific *BRCA1/2* genetic testing, particularly for triple‐negative BC patients known for their high prevalence in Tanzania. This will, in turn, greatly contributes to provide effective therapeutic strategies.

## Graphical abstract

This study investigated the prevalence and spectrum of germline single nucleotide variant/insertion and deletion (SNV/indel), and copy number variations (CNVs) in *BRCA1/2* among Tanzanian breast cancer patients, and evaluated the associations of identified variants with patient’s socio‐demographic and histopathological characteristics. Next‐generation sequencing results showed that SNV/indel were detected in 6% of patients, and CNVs was not detected in any patient. Our findings indicate that *BRCA1/2* pathogenic variants may well contribute to breast cancer incidence in Tanzania. Thus, the identification of frequent variants in *BRCA1/2* genes will enable implementation of rapid, inexpensive population‐specific *BRCA1/2* genetic testing particularly for triple‐negative breast cancer patients known for their high prevalence in Tanzania.

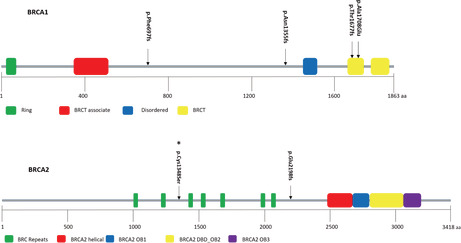

## 1 INTRODUCTION

Breast cancer is the most commonly diagnosed cancer among women worldwide, and the fifth leading cause of cancer deaths estimated at 685,000 in 2020.^1^ In developing countries including the vast majority of Sub‐Saharan Africa (SSA), BC is the second most common female malignancy after cervical cancer.^2^ In Tanzania, the incidence rates of BC are on the rise over the past two decades and were predicted to increase by over 80% by 2030.^3^, ^4^ About half of women diagnosed with BC in Tanzania die of the disease as majority of them present with advanced stage (stage III or IV) where treatment is less effective, expensive, and the outcomes are poor.^3^

Majority of the BC cases are sporadic with no cancer history in the first or second‐degree relatives. However, about 5%–10% of BC cases have a hereditary background and can be passed on from generation to generation.^5^ Breast cancer genes, *BRCA1/2*, are tumor suppressor genes located on chromosomes 17q21 and 13q12‐13, respectively, are mainly responsible for the maintenance of genome integrity through homologous DNA repair and control of cell cycle. The two genes are the major hereditary BC and/or ovarian cancer (OC) predisposition genes, reported for the first time over two decades ago.^6^, ^7^ Individuals harboring pathogenic variants in *BRCA1/2* have an elevated lifetime risk of developing BC, OC, prostate cancer and pancreatic cancer.^8^, ^9^, ^10^ Scholars describe women with pathogenic variants in *BRCA1* to have about 72% and 44% cumulative risk of BC and OC, respectively. Meanwhile, those with pathogenic variants in *BRCA2* have about 69% and 17% cumulative risk of BC and OC, respectively, before the age of 80.^11^ It is worth noting that not all pathogenic *BRCA1/2* variant carriers develop cancer in a lifetime. Therefore, healthy individual carriers may be recommended to undergo intensified cancer surveillance, use chemoprevention prophylaxis, or consider prophylactic surgery as a means of reducing risks of BC, OC, and other cancers. By undergoing said measures, the disease can be prevented or detected at a very early stage.^2^

Thousands of the germline *BRCA1/2* variants are known to date, and the numbers continue to increase with the majority of them being SNV/indel occurring over the entire length of the genes. Detection of these SNV/indel variants together with large deletion and duplications also known as copy number variations (CNVs) is currently more feasible and cost‐effective following the invention of next‐generation sequencing (NGS). This enables simultaneous detection of both variants using a single platform and workflow, which provides high accuracy and shorter turnaround time.^12^, ^13^ Although the CNVs are seldom reported, they contribute a substantial fraction of germline pathogenic *BRCA1/2* variants of about 4%–28%. The CNVs are more common in *BRCA1* than in *BRCA2* due to the presence of high densities of *Alu* sequences and homologous recombination events between *BRCA1* and its pseudogenes.^14^, ^15^

Genetic testing for pathogenic *BRCA1/2* SNV/indel and CNVs has emerged as a new weapon to fight against BC and is gaining more implications in clinical management as well as in general public health.^16^ BC patients confirmed of carrying pathogenic *BRCA1/2* variants have a chance of benefiting from advanced personalized BC treatments such as the PARP inhibitors.^17^ However, *BRCA1/2* genetic testing and counseling services are not available in majority of oncology centers in SSA. Barriers to implementation of genetic testing and counseling services in SSA include but not limited to high costs of establishing and continuous running of testing services, and limited capacity among health services providers. In Tanzania, *BRCA1/2* genetic testing and counseling are not available in either public or private oncology centers. Patients who are recommended to undergo these services may be referred to the Republic of South Africa or overseas.

Studies across different populations report the prevalence and spectrum of germline SNV/indel and CNVs in *BRCA1/2*. Despite the commonalities in study designs, varying results have been reported due to differences in study population and geographic locations where they are undertaken.^18^ The identification of germline *BRCA1/2* population‐specific variants is a crucial milestone toward establishing and incorporating *BRCA1/2* genetic testing and counseling into clinical practice. The *BRCA1/2* information regarding the prevalence and spectrum is limited in the fast‐growing African populations, with a few findings from Northern Africa,^19^, ^20^, ^21^ Southern Africa,^22^, ^23^ and Western Africa.^24^, ^25^ In an attempt to understanding the prevalence and spectrum of germline *BRCA1/2* variants in indigenous populations of Eastern Africa, we initiated a study that analyzed the prevalence of germline SNV/indel pathogenic variants and CNVs in *BRCA1/2* in Tanzanian BC patients unselected for age at diagnosis and family history of cancer. To achieve this goal, we employed NGS technology to analyze DNA samples of 100 BC patients. In addition, we employed Sanger sequencing, and multiplex ligation‐dependent probe amplification (MLPA) as confirmatory assays for SNV/indel pathogenic variants, and CNVs, respectively. Furthermore, we determined the association of identified variants with the patients' clinico‐histopathological and socio‐demographic characteristics. To the best of our knowledge, this is the first genetic study employing NGS to analyze the germline *BRCA1/2* variants among BC patients at the only tertiary cancer‐specialized public facility in Tanzania.

## 2 MATERIALS AND METHODS

### 2.1 Study population

A total of 100 BC patients participated in the present study. The BC patients reported here were recruited between September 2019 and May 2021 at the Ocean Road Cancer Institute (ORCI): the only tertiary cancer‐specialized public hospital located at the shores of the Indian Ocean in Dar es Salaam, Tanzania. The ORCI receives BC referral cases from all regions of the country for chemotherapy, immunotherapy, endocrine therapy, radiotherapy, and palliative care. Further details about the ORCI facility are described elsewhere.^26^, ^27^ To be eligible for this study, patients had to fulfill the following inclusion criteria: being indigenous Tanzanian, and having a complete histopathological report that shows the estrogen receptor (ER), progesterone receptor (PR), and human epidermal growth factor receptor‐2 (HER‐2) statuses. The exclusion criteria were: being non‐indigenous Tanzanian, failure/denial to sign a consent form, and having incomplete histopathological report in the hospital medical file. Face‐to‐face interviews were conducted to capture patient's socio‐demographic characteristics, family history of cancer, and reproductive behavior information. Clinical data on BC presentation, diagnosis, and staging were extracted from patients' hospital records. The study protocol was approved by the Ethics Committee of the Tanzania National Institute for Medical Research (NIMR) permit no. *NIMR/HQ/R*.*8a/Vol*. *IX/3255*, and the ORCI Institution Review Board permit no. *10/Vol/XX/16*. A written informed consent form from each participant was obtained.

### 2.2 Blood sample collection and DNA isolation

Peripheral blood sample (5 ml) was collected from the antecubital vein of each study participant and stored in EDTA tubes until used for DNA extraction. Genomic DNA was manually extracted from blood leucocytes using High Pure PCR Template Preparation Kit no. 11796828001 (Roche Life Science) following manufacturer's recommendations. The DNA integrity was assessed by running a 0.8% agarose gel electrophoresis and viewed under UV light (Gel Doc™ XR+, BIORAD, USA). The quantity and quality were determined using NanoDrop™ 2000 spectrophotometer (Thermo Scientific). The DNA yield minimum of 5 ng/μl and the ranges of OD between 1.8 and 2 at A260 nm/280 nm were considered ideal for downstream processes.

### 2.3 *BRCA1/2* SNV/indel detection by next‐generation sequencing

*BRCA1/2* variants on DNA were examined with NGS technique using the Multiplicom BRCA MASTR Plus kit (Agilent Technologies) following the manufacturer's instructions. In brief, for each patient, 280 ng of genomic DNA was used to perform multiplex polymerase chain reactions (PCR) to amplify targeted regions. Thereafter, the multiplexed tagged regions were purified using the Agencourt AMPure XP Beads (Beckman Coulter) and quantified using the Qubit Fluorimeter. An equal volume of multiplexed tagged reactions from the same sample was taken and combined in LoBind Eppendorf and a sample pool was formed. The sample pool concentration was measured in the Qubit Fluorimeter. After determining the appropriate concentration, sequence analysis was performed using Illumina NextSeq (Illumina® NextSeq™ Illumina).

### 2.4 Bioinformatics analysis

Generated raw data from NGS analysis were uploaded to the SOPHIA DDM™ (Sophia Genetics SA) platform and analyzed referring to the Hg19 reference genome (Genome Reference Consortium human build 37, GRCh37). Variants and CNVs analyses of the samples are listed on SOPHIA DDM™. Genetic variants classification was done according to the guidelines of the American College of Medical Genetics and Genomics (ACMG) by a five‐tier system (a pathogenic, likely pathogenic, variant of unknown significance (VUS), likely benign, or benign).^28^ The NM_007294.4 and NM_000059.3 cDNA sequences served as reference transcripts for *BRCA1* and *BRCA2*, respectively. The presence of variants was evaluated using databases such as Human Genome Mutation Database (HGMD) professional, single nucleotide polymorphism database (dbSNP), and public archive of interpretations of clinically relevant variants (ClinVar) for clinical importance after classification. All SNV/indel pathogenic variants in *BRCA1/2* and CNVs identified by NGS were confirmed via Sanger sequencing and MLPA assay, respectively.

### 2.5 SNV/indel confirmation by Sanger sequencing

Sanger sequencing was performed to confirm the NGS‐detected germline *BRCA1/2* pathogenic variants. Sequencing primers (Supplementary Table S1) flanking the identified pathogenic variants were designed using Primer 3.0. The PCR products were purified using an ExoSAP‐IT™ PCR Product Cleanup Reagent (Applied Biosystems), as per the manufacturer's instructions. Sequencing reactions were performed using BigDye® Terminator v3.1 Cycle Sequencing Kit (Applied Biosystems) on an ABI 96‐capillary 3130xl DNA Analyzer (Applied Biosystems), followed by sequences analysis using MEGA‐X v6 software.

### 2.6 CNVs confirmation by MLPA assay

The sample suspected of having CNV in *BRCA1* by NGS analysis was confirmed by MLPA assay using the SALSA P002‐D10720 (MRC‐Holland) following manufacturer's recommendations. Fragment analysis of amplified DNA was run on an ABI‐3130XL genetic analyzer (Applied Biosystems). Ten normal controls were included as references in the MLPA run. Evaluation of MLPA results was performed in a Coffalyser software v.140721.1958. All peak heights were normalized, where a ratio between 0.7 and 1.3 was regarded as normal.

### 2.7 Statistical analyses

The data were analyzed in Statistical Package for the Social Sciences 25.0 (IBM SPSS, Inc.). Differences between groups about clinico‐pathological, anthropometric, socio‐demographic, and reproductive characteristics were examined using chi‐square (χ2) tests. The results were expressed as the mean ± standard deviation, or percentage, wherever appropriate. The *p*‐value ˂0.05 was considered statistically significant.

## 3 RESULTS

### 3.1 Characteristics of the study cohort

The 100 BC patients analyzed in the present series were unselected for family history of cancer and age at diagnosis. The mean age at BC diagnosis was 44.04 ± 11.54 years. Fifty‐two percent of patients had carcinoma of the left breast, 46% of the right breast, and 2% had carcinoma of both breasts. Majority (91%) of the participants were diagnosed with invasive ductal carcinoma of no specific type (IDC‐NST). Luminal‐A and triple‐negative subtypes of BC were of comparable proportion, each accounting for 36% and 35%, respectively. A few (18%) of the participants had a history of BC and other cancers in their families. The socio‐demographic and clinico‐histopathological features of the cohort are given in Table 1.

**TABLE 1:** Socio‐demographic and clinico‐histopathological characteristics of the study population

Characteristic	Number of patients, *n* (%)
Age at breast cancer diagnosis (Mean ± SD)	44.04 ± 11.54
˂40	40 (40%)
40–49	39 (39%)
≥50	21 (21%)
Patient's origin	
Central zone	12 (12%)
Eastern zone	16 (16%)
Lake zone	9 (9%)
Northern zone	24 (24%)
Southern highlands zone	18 (18%)
Southern zone	14 (14%)
Western zone	7 (7%)
BMI	
Underweight (below 18.5)	3 (3%)
Normal (18.5–24.9)	40 (40%)
Overweight (25.0–29.9)	23 (23%)
Obese (30.0+)	34 (34%)
Family history of cancer	
Yes	18 (18%)
No	82 (82%)
TNM pathological stage	
Stage I	0 (0%)
Stage II	14 (14%)
Stage III	56 (56%)
Stage IV	30 (30%)
Histological type	
IDC‐NST	91 (91%)
ILC	6 (6%)
Others	3 (3%)
Laterality	
Left	52 (52%)
Right	46 (46%)
Bilateral	2 (2%)
ER status	
Positive	49 (49%)
Negative	51 (51%)
PR status	
Positive	31 (31%)
Negative	69 (69%)
HER‐2 status	
Positive	29 (29%)
Negative	71 (71%)
Molecular subtype	
Luminal‐A	36 (36%)
Luminal‐B	17 (17%)
Triple‐negative	35 (35%)
HER‐2 enriched	12 (12%)
Menopausal status	
Post‐menopause	38 (38%)
Pre‐menopause	62 (62%)
Breastfeeding	
Yes	83 (83%)
No	17 (17%)
Contraceptives use	
Yes	45 (45%)
No	55 (55%)
Ever been pregnant	
Yes	90 (90%)
No	10 (10%)
Alcohol consumption	
Yes	18 (18%)
No	82 (82%)
Smoke exposure	
Yes	0 (0%)
No	100 (100%)

Abbreviations: BMI, Body mass index; ER, Estrogen receptor; HER‐2, Human epidermal growth factor receptor‐2; IDC‐NST, Invasive ductal carcinoma of no specific type; ILC, Invasive lobular carcinoma; PR, Progesterone receptor; SD, Standard deviation; TNM, Tumor node metastasis.

### 3.2 SNV/indel pathogenic variants of the study cohort

**FIGURE 1:**
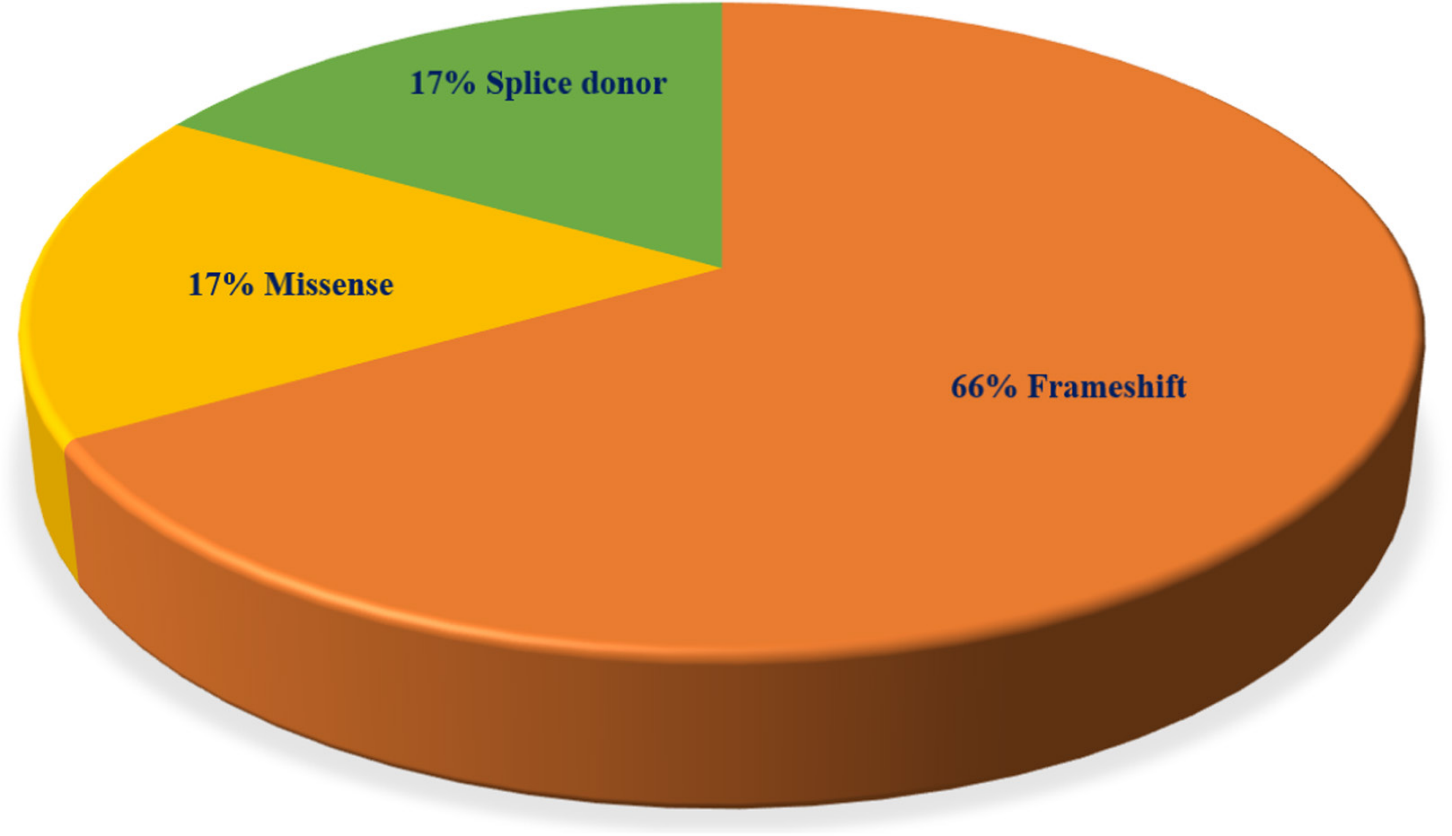
Proportion of germline SNV/indel *BRCA1/2* pathogenic variants detected in 100 Tanzanian breast cancer patients. The figure was generated using GraphPad Prism v. 8.0 (GraphPad Software Inc., San Diego, CA, USA).

**FIGURE 2:**
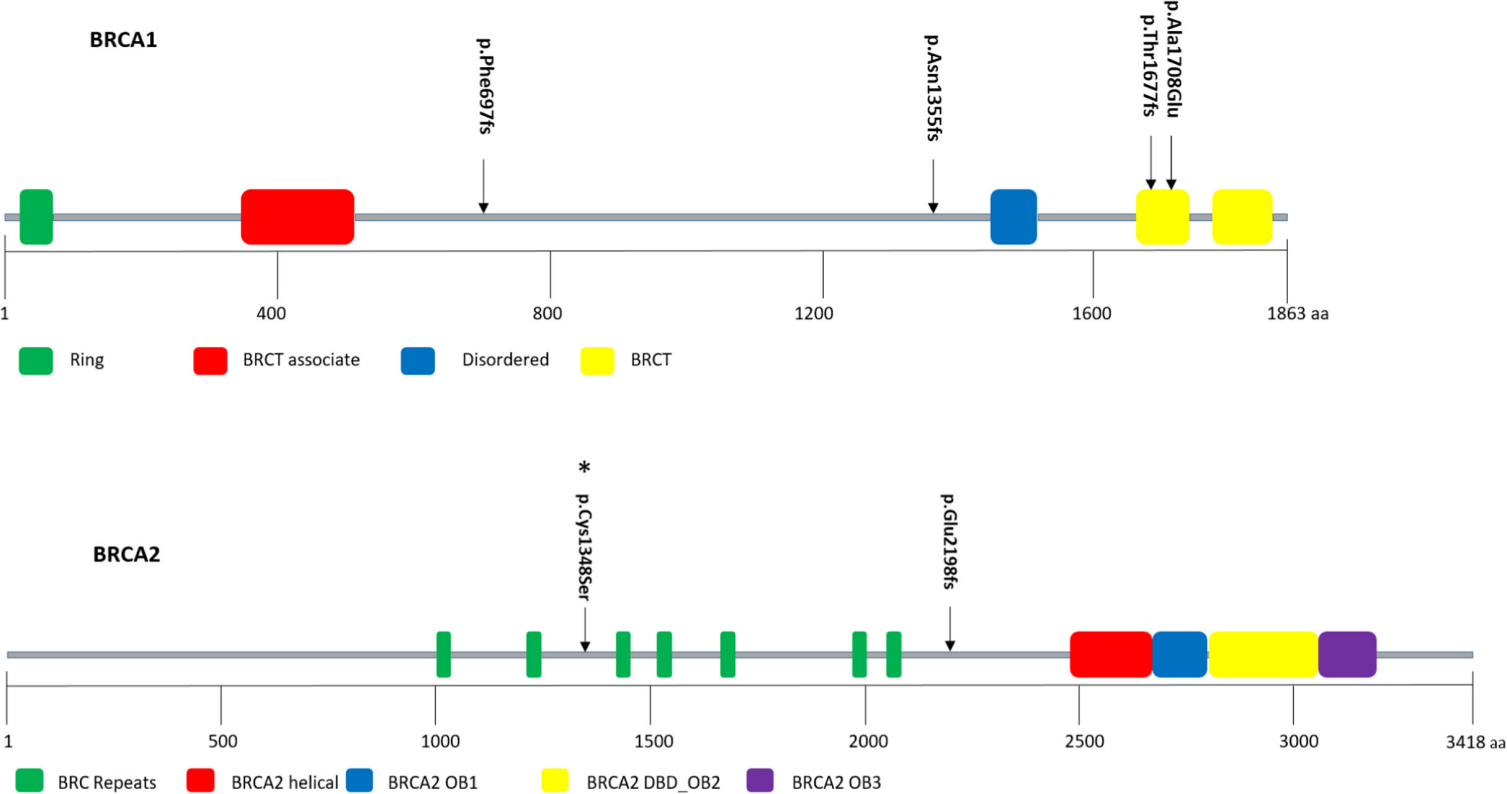
SNV/indel pathogenic variants and VUS (*) detected in Tanzanian breast cancer patients displayed along the BRCA1/2 proteins. BRCA1 variants are c.2090del (p.Phe697fs), c.4065_4068del (p.Asn1355fs), c.5030_5033del (p.Thr1677fs), c.5123C>A (p.Ala1708Glu), and a splice donor c.212 + 1G>A (protein unknown, hence not shown). BRCA1 domains are Ring/Zinc finger (green), BRCT associate/Serine‐rich (red), Disordered region (blue), and BRCT (yellow). BRCA2 variant is c.6591_6592del (p.Glu2198fs), and a VUS c.4042T>A (p.Cys1348Ser). BRCA2 domains are BRC repeats (green), BRCA2 helical (red), BRCA2 oligosaccharide binding: BRCA2 OB1 (blue), BRCA2 oligosaccharide/oligonucleotide binding: BRCA2 DBD_OB2 (yellow), and BRCA2 oligosaccharide binding: BRCA2 OB3 (purple).

In our analysis, we detected 6% SNV/indel pathogenic variants from unrelated BC patients. Five percent of these variants were identified in *BRCA1*, and 1% in *BRCA2*. Of these six SNV/indel pathogenic variants, four were frameshift, one was a missense, and the last one was a splice donor (Figure 1). With the exception of the splice donor variant, the rest of SNV/indel *BRCA1/2* pathogenic variants are illustrated on the protein structures in Figure 2. All the pathogenic variants identified in our study were verified in a ClinVar database and are detailed in Table 2 and Supplementary Figure S1. Five of six *BRCA1/2* SNV/indel pathogenic mutation carriers were diagnosed with triple‐negative subtype, only one patient was diagnosed with luminal‐B subtype, and five of six patients were diagnosed with BC at ≤45 years.

**TABLE 2:** Germline *BRCA1/2* SNV/indel pathogenic variants identified in Tanzanian breast cancer patients and their clinico‐pathological characteristics

Gene	Variant at DNA level	Variant at protein level	Exon	Variant type	Molecular consequences	Clinical significance (ClinVar)	dbSNP ID	Age at breast cancer diagnosis	TNM stage	Molecular subtype	Family history of cancer
*BRCA1*	c.212 + 1G>A	—	4	SNV	Splice donor	Pathogenic	rs80358042	32	III	Triple‐negative	No
*BRCA1*	c.4065_4068del	p.Asn1355fs	10	Deletion of TCAA	Frameshift	Pathogenic	rs80357508	34	Unknown	Triple‐negative	Yes: BC, Paternal Aunt, dx?, dcd 55
*BRCA1*	c.2090del	p.Phe697fs	10	Deletion of A	Frameshift	Pathogenic	rs886039996	40	II	Luminal‐B	Yes: BC, Maternal Aunt, dx 50, dcd 52
*BRCA1*	c.5030_5033del	p.Thr1677fs	16	Deletion of CTTA	Frameshift	Pathogenic	rs80357580	53	III	Triple‐negative	No
*BRCA1*	c.5123C>A	p.Ala1708Glu	17	SNV	Missense	Pathogenic	rs28897696	29	II	Triple‐negative	No
*BRCA2*	c.6591_6592del	p.Glu2198fs	11	Deletion of TG	Frameshift	Pathogenic	rs80359605	45	III	Triple‐negative	No

Abbreviations: BC, Breast cancer; dbSNP, Single nucleotide polymorphism database; dcd, Age at death; dx, Age at diagnosis; Indel, Insertion/deletion; SNV, Single nucleotide variation; TNM, Tumor node metastasis.

### 3.3 Spectrum of *BRCA1* */2* pathogenic variants identified among the study cohort

Five different germline pathogenic variants detected in *BRCA1* in the current series were named with reference to sequences of accession number NM_007294.4 (DNA) and NP_009225.1 (protein). The first SNV pathogenic variant identified in this study was the c.212 + 1G>A. The name of this variant at protein level is still unknown. This is a spice donor variant designated as IVS5 + 1G>A in some literature and BIC database. The variant results from the substitution of glycine by alanine after exon 4 of the *BRCA1*. A patient identified to harbor this pathogenic variant in our cohort was a single lady diagnosed with BC at age of 32. This patient had no family history of cancer and presented with IDC‐NST of the left breast. Immunohistochemistry analysis of her tumor showed a triple‐negative status.

**FIGURE 3:**
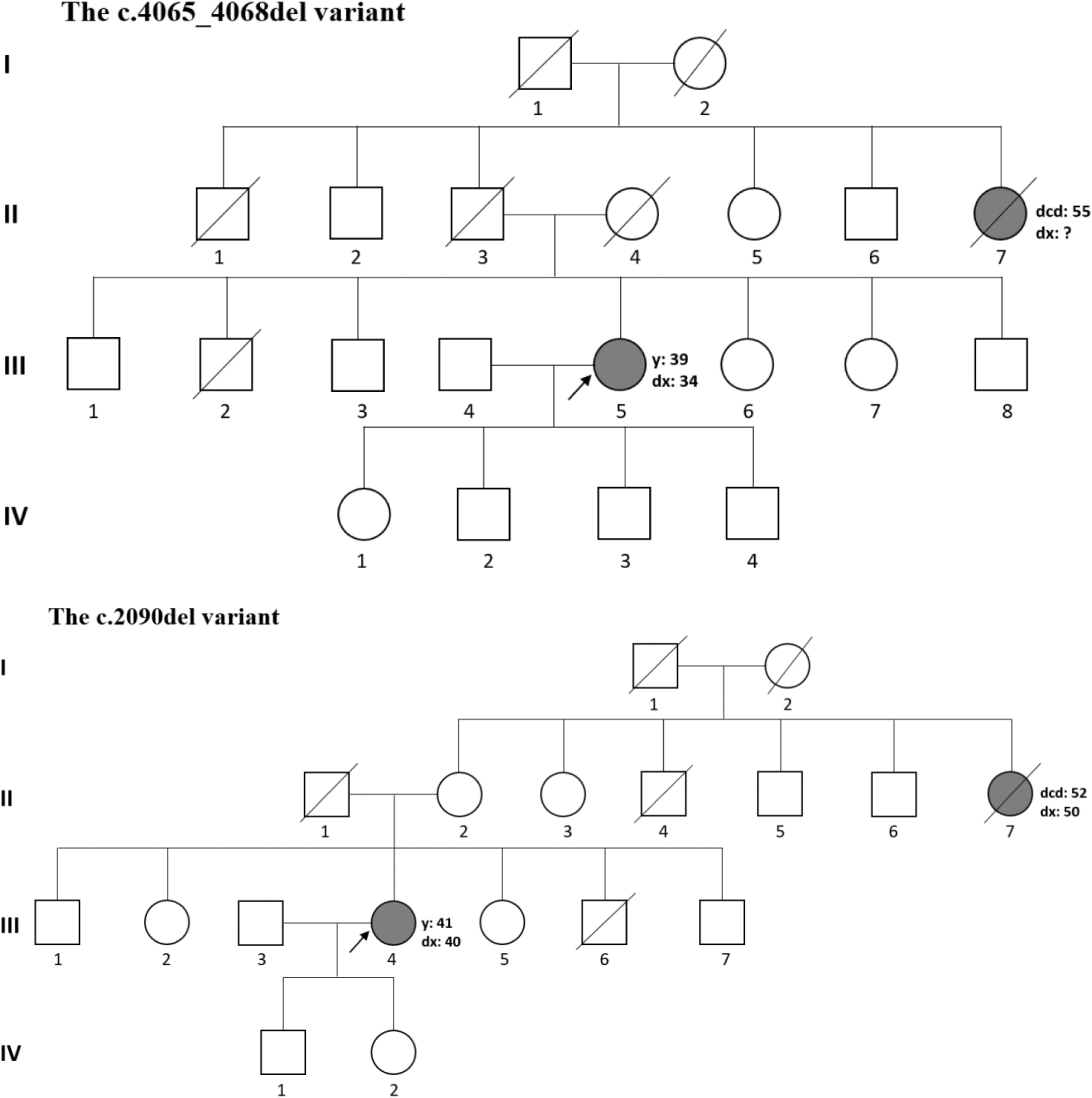
Family trees of Tanzanian breast cancer patients with a family history of cancer that were identified harboring the c.2090del, and the c.4065_4068del pathogenic variants of *BRCA1*. Roman numerals indicate generations. Squares and circles indicate male and female, respectively. Members affected by breast cancer are indicated with a shading. The arrow indicates the proband. A diagonal slash denotes a deceased member. Age at breast cancer diagnosis, at interview, and at death are denoted by dx, y, and dcd, respectively.

The c.4065_4068del (p.Asn1355fs) was the first indel pathogenic *BRCA1* variant identified in this study. This is a frameshift variant arising as a consequence of deletion of TCAA residues in exon 10 (c.4065_4068delTCAA). The variant was detected in a patient who presented with IDC‐NST of the right breast at age of 34. This patient was diagnosed with triple‐negative BC and had her paternal aunt died of BC at 55 years (Figure 3).

The second indel *BRCA1* pathogenic variant was c.2090del (p.Phe697fs). This is a frameshift variant due to deletion of adenine residue at position 2090 in exon 10 of a coding DNA (cDNA), causing an alteration of amino acid sequence from position 697 in a corresponding protein, that consequently leads to premature termination of translation. We identified this variant in a patient diagnosed with IDC‐NST of the left breast at the age of 40. Her tumor was characterized as ER‐positive, PR‐positive, and HER‐2‐positive (Luminal‐B subtype). The patient harboring this variant reported a family history of BC in her maternal aunt who died at 52 years (Figure 3).

The third indel pathogenic variant detected in *BRCA1* was c.5030_5033del (p.Thr1677fs). This is a frameshift variant located in exon 16 of *BRCA1* resulting from a deletion of CTTA nucleotide residues at position 5030 to 5033 of the cDNA, thus, creating a premature translational stop signal: p.Thr1677Ilefs*2. This variant locates within the BRCT domain of the BRCA1 protein (Figure 2) and was detected in a patient diagnosed with triple‐negative BC at 53 years of age. The patient had no family history of cancer and presented with IDC‐NST of the right breast.

The c.5123C>A (p.Ala1708Glu) variant was the second SNV *BRCA1* pathogenic variant identified in the patients group. This is a missense variant arising as a result of substitution of cytosine residue by a thiamine at position 5123 of exon 17 of the cDNA. The substitution results in a change of alanine to glutamine residue at position 1708 of a BRCA1 protein. This variant is located in the BRCT domain of the BRCA1 protein (Figure 2), a region that interacts with several other proteins. The BC patient harboring this variant in our series was diagnosed with ILC of the left breast at the age of 29. The tumor immunohistochemistry results revealed a triple‐negative status. The patient was an overweight, non‐smoker, non‐drinker, nulliparous married female.

The c.6591_6592del (p.Glu2198fs) was the only indel pathogenic variant detected in *BRCA2* in this study. This variant is a deletion of two nucleotides (TG) in exon 11 of the cDNA at position 6591 to 6592. The deletion causes a frameshift which changes a glutamate at position 2198 by creating a premature stop codon at position 4 of the new reading frame. This variant results in a premature termination codon, predicted to cause an absence or a truncated protein. In our cohort, this variant was detected in a patient diagnosed with IDC‐NST of the right breast at age of 45. The patient was a peasant and had no family history of cancer. The immunohistochemistry analysis revealed a triple‐negative status of her tumor.

### 3.4 VUS and benign variants of *BRCA1/2* identified among the study cohort

Among the 100 successfully sequenced patients in the current series, only one patient was identified to have a VUS. The identified VUS: c.4042T>A (p.Cys1348Ser) (Figure 2) is a SNV involving substitution of thiamine nucleotide by an adenine in exon 11 of *BRCA2*, resulting in substitution of cytosine to serine at position 1348 of the BRCA2 protein. The patient identified to harbor this VUS was diagnosed with triple‐negative BC at a young 29 years of age. Additionally, we identified 19 and 17 homozygous variants in *BRCA1* and *BRCA2*, respectively, in our cohort. All of the 36 variants detected had been reported elsewhere and exist in the ClinVar database where they are accorded a benign clinical significance status (Table 3).

**TABLE 3:** Germline *BRCA1/2* VUS and benign variants identified in Tanzanian breast cancer patients

Gene	Variant at DNA level	Variant at protein level	Exon	Variant type	Molecular consequences	Clinical significance (ClinVar)	dbSNP ID	Number of carriers
*BRCA1*	c.19‐115T>C	—	1	SNV	—	Benign	rs3765640	1
*BRCA1*	c.442‐34C>T	—	6	SNV	—	Benign	rs799923	1
*BRCA1*	c.548‐58del	—	7	Deletion T	—	Benign	rs8176144	1
*BRCA1*	c.1067A>G	p.Gln356Arg	10	SNV	Missense	Benign	rs1799950	1
*BRCA1*	c.1843TCT	p.Ser616del	10	3 bp Microsatellite	In frame mutation	Benign	rs80358329	1
*BRCA1*	c.2077G>A	p.Asp693Asn	10	SNV	Missense	Benign	rs4986850	1
*BRCA1*	c.2082C>T	p.Ser694=	10	SNV	Synonymous	Benign	rs1799949	3
*BRCA1*	c.2311 T>C	p.Leu771=	10	SNV	Synonymous	Benign	rs16940	1
*BRCA1*	c.2612C>T	p.Pro871Leu	10	SNV	Missense	Benign	rs799917	73
*BRCA1*	c.3113A>G	p.Glu1038Gly	10	SNV	Missense	Benign	rs16941	1
*BRCA1*	c.3548A>G	p.Lys1183Arg	10	SNV	Missense	Benign	rs16942	3
*BRCA1*	c.4097‐141A>C	—	10	SNV	—	Benign	rs799916	1
*BRCA1*	c.4308 T>C	p.Ser1436=	12	SNV	Synonymous	Benign	rs1060915	1
*BRCA1*	c.4485‐63C>G	—	13	SNV	—	Benign	rs273900734	1
*BRCA1*	c.4837A>G	p.Ser1613Gly	15	SNV	Missense	Benign	rs1799966	3
*BRCA1*	c.4987‐68A>G	—	15	SNV	—	Benign	rs8176234	1
*BRCA1*	c.4987‐92A>G	—	15	SNV	—	Benign	rs8176233	1
*BRCA1*	c.5152 + 66G>A	—	16	SNV	—	Benign	rs3092994	1
*BRCA1*	c.5152 + 85del	—	16	Deletion T	—	Benign	rs8176259	3
*BRCA2*	c.425 + 67A>C	—	3	SNV	—	Benign	rs11571610	1
*BRCA2*	c.426‐89T>C	—	3	SNV	—	Benign	rs3783265	1
*BRCA2*	c.631 + 183T>A	—	5	SNV	—	Benign	rs3752451	1
*BRCA2*	c.865A>C	p.Asn289His	9	SNV	Missense	Benign	rs766173	1
*BRCA2*	c.1114A>C	p.Asn372His	10	SNV	Missense	Benign	rs144848	1
*BRCA2*	c.1365A>G	p.Ser455=	10	SNV	Synonymous	Benign	rs1801439	1
*BRCA2*	c.2229 T>C	p.His743=	11	SNV	Synonymous	Benign	rs1801499	1
*BRCA2*	c.2971A>G	p.Asn991Asp	11	SNV	Missense	Benign	rs1799944	2
*BRCA2*	c.3807 T>C	p.Val1269=	11	SNV	Synonymous	Benign	rs543304	2
*BRCA2*	c.4042 T>A	p.Cys1348Ser	11	SNV	Missense	VUS	rs199863034	1
*BRCA2*	c.4563A>G	p.Leu1521=	11	SNV	Synonymous	Benign	rs206075	88
*BRCA2*	c.6513G>C	p.Val2171=	11	SNV	Synonymous	Benign	rs206076	87
*BRCA2*	c.7397 T>C	p.Val2466Ala	14	SNV	Missense	Benign	rs169547	87
*BRCA2*	c.7435 + 53C>T	—	14	SNV	—	Benign	rs11147489	1
*BRCA2*	c.7806‐14 T>C	—	15	SNV	—	Benign	rs9534262	1
*BRCA2*	c.8755‐66 T>C	—	20	SNV	—	Benign	rs4942486	1
*BRCA2*	c.10234A>G	p.Ile3412Val	27	SNV	Missense	Benign	rs1801426	2
*BRCA2*	c.*105A>C	—	27	SNV	3' UTR	Benign	rs15869	1

Abbreviations: dbSNP, Single nucleotide polymorphism database; SNV, Single nucleotide variation; UTR, Untranslated region; VUS, Variant of uncertain significance.

### 3.5 Copy number variation

MLPA assay was applied to confirm the CNV results obtained after NGS analysis. It was confirmed that none of the patients harbored CNVs in either *BRCA1* or *BRCA2* in this cohort.

### 3.6 Associations between germline *BRCA1/2* variant status and patients' clinicohistopathological characteristics

The associations of germline *BRCA1/2* pathogenic variants of 100 BC patients with their clinicohistopathological characteristics are summarized in Table 4. *BRCA1/2* variant carriers were significantly more likely to be triple‐negative subtype of BC than non‐*BRCA1/2* carriers (*p* = 0.019). No significant association was found in the status of the age at BC diagnosis, family history of cancer, histological type, ER, PR, HER‐2, menopausal, and BMI.

**TABLE 4:** Associations of detected germline *BRCA1/2* pathogenic variants with their clinicohistopathological characteristics

Characteristic	*BRCA1/2* pathogenic variant carriers	*BRCA1/2* variant non‐carriers	*p*‐value
Age at breast cancer diagnosis			
˂40	3 (50%)	37 (39.4%)	0.684
40–49	2 (33.3%)	37 (39.4%)	
≥50	1 (16.7%)	20 (21.3%)	
Histological type			
IDC‐NST	5 (83.3%)	86 (91.5%)	0.695
ILC	1 (16.7%)	5 (5.3%)	
Others	0 (0%)	3 (3.2%)	
Family history of cancer			
Yes	2 (33.3%)	16 (17%)	0.294
No	4 (66.7%)	78 (83%)	
ER status			
Positive	1 (16.7%)	48 (51.1%)	0.112
Negative	5 (83.3%)	46 (48.9%)	
PR status			
Positive	1 (16.7%)	30 (31.9%)	0.393
Negative	5 (83.3%)	64 (68.1%)	
HER‐2 status			
Positive	1 (16.7%)	28 (29.8%)	0.437
Negative	5 (83.3%)	66 (70.2%)	
Molecular subtype			
Luminal‐A	0 (0%)	36 (38.3%)	**0.019**
Luminal‐B	1 (16.7%)	16 (17%)	
Triple‐negative	5 (83.3%)	30 (31.9%)	
HER‐2 enriched	0 (0%)	12 (12.8%)	
Menopausal status			
Pre‐menopause	3 (50%)	59 (62.8%)	0.413
Post‐menopause	3 (50%)	35 (37.2%)	
BMI			
Underweight (˂18.5)	0 (0%)	4 (4.3%)	0.871
Normal (18.5–24.9)	3 (50%)	37 (39.4%)	
Overweight (25.0–29.9)	1 (16.7%)	20 (21.3%)	
Obese (≥30.0)	2 (33.3%)	33 (35.1%)	

*Note*: The values are calculated using the chi‐square test, data are given in percentages. The *p*‐value ˂0.05 was considered statistically significant (in bold).

Abbreviations: BMI, Body mass index; ER, Estrogen receptor; HER‐2, Human epidermal growth factor receptor‐2; IDC‐NST, Invasive ductal carcinoma of no specific type; ILC, Invasive lobular carcinoma; PR, Progesterone receptor.

## 4 DISCUSSION

In SSA countries, only a few studies have explored hereditary BC through genetic analysis of breast cancer susceptibility genes such as *BRCA1*, *BRCA2*, and others. Access to genetic testing remains a challenge in many SSA countries and is not a component of routine BC management practices. The germline *BRCA1/2* pathogenic variants are widely reported to play a substantial role in BC predisposition in transitioned countries, while very little is known from developing countries of SSA, despite the increasing cases from year to year.^1^ To the best of our knowledge, the contribution of germline *BRCA1/2* pathogenic variants to BC incidences among the indigenous Tanzanian women has never been studied. Therefore, this is the first study reporting the frequency and spectrum of germline SNV/indel and CNVs in *BRCA1/2* among the Tanzanian BC patients unselected for family history of cancer and age at diagnosis. Also, the study presents the associations of identified *BRCA1/2* pathogenic variants with the patients' clinico‐histopathological features.

In the present study, we applied NGS, Sanger sequencing, and MLPA to screen germline SNV/indel and CNVs in *BRCA1/2* among 100 BC patients enrolled at the only tertiary cancer‐specialized public hospital located in business capital, Dar es Salaam, Tanzania. The design of this study enabled us to recruit a cohort of patients reflecting the ideal representation of the indigenous Tanzanian population from all regions of the country. The coding exons and intron/exon junctions of *BRCA1/2* were carefully sequenced. The mean age at diagnosis of our cohort was 44.04 ± 11.54 years. Our cohort was younger compared to previous BC cohorts studied in Tanzania, in which two independent studies reported that an average age of BC patients at diagnosis was around 51 years.^4^, ^29^

We observed an overall 6% frequency for germline SNV/indel pathogenic variants and CNVs in *BRCA1/2*. Among them, 5% of variants were detected in *BRCA1* and were either a SNV or a deletion. The remaining 1% variant was a deletion detected in *BRCA2*. All SNV/indel pathogenic variants (c.212 + 1G>A, c.4065_4068del, c.2090del, c.5030_5033del, c.5123C>A, and c.6591_6592del) were detected in six unrelated patients. All the pathogenic variants identified in our cohort were mentioned in ClinVar database and are given in Table 2. Despite the fact that our cohort was not selected for age at diagnosis, the prevalence of germline *BRCA1/2* pathogenic variants observed does not differ significantly from that observed in the Rwandan BC patients diagnosed with the disease before 35 years.^12^ Our findings also agree with other scholars' findings from unselected Chinese BC patients,^30^ Sweden,^31^ and Bahrain that analyzed 25 patients selected for early onset of the disease and family history of cancer.^32^ However, the prevalence observed in our cohort (6%) is lower compared to that reported in Uganda and Cameroon combined,^33^ Morocco,^34^ and Mexico.^35^ The prevalence of germline *BRCA1/2* pathogenic variants between studies may possibly vary by race/ethnicity of population, study sample size, and different enrollment criteria.

In our series, we found that *BRCA1* has a higher frequency of germline pathogenic variants than *BRCA2* (5 variants vs 1 variant). Our findings are in concordance with some studies^34^, ^35^, ^36^ and differ from studies that reported a higher frequency of *BRCA2* over *BRCA1*.^12^, ^37^ A few studies have reported co‐dominance in their overall analysis.^31^, ^33^ These discrepancies call for more consistent large‐scale *BRCA1/2* studies across different populations to build a stronger conclusion regarding the prevalence and spectrum of pathogenic variants in the *BRCA1/2*.

*BRCA1* variants occur mainly in four domains of the corresponding protein: N‐terminus ring domain (exon 1–6), BRCT associate domain (exon 10–12), and two BRCT domains at the C‐terminus (exon 15–23) (Figure 2). Variants in these domains may disrupt the DNA repair, control of cell cycle, and other functions of the BRCA1 protein.^30^ In the present study, identified pathogenic variants were located in exons 4, 10, 16, and 17 of the *BRCA1*. Exon 10 had two pathogenic variants, whereas the other exons had one pathogenic variant each. *BRCA2* codes for a protein made up of helical domain (for DNA‐binding), three oligonucleotide/oligosaccharide binding domains that encompass a tower domain, and seven to eight BRC repeats. In the present study, the only detected pathogenic variant occurred in a region of exon 11 of *BRCA2* outside the domains of a BRCA2 protein (Figure 2). Variant in this exon may disrupt the interaction of BRCA2 with other proteins such RAD51 and TP53 that work together to mediate DNA damage repair.^38^

We did not detect any new/novel *BRCA1/2* variant specific for the Tanzanian population. Thus, the spectrum of the germline *BRCA1/2* variants observed in our cohort does not differ from other populations. The germline *BRCA1/2* pathogenic variants are widely reported among families with a strong history of breast/ovarian cancer,^10^, ^39^, ^40^ cohort of early‐onset BC patients,^41^ triple‐negative BC patients,^42^ pancreatic cancer patients,^9^ and among patients with prostate cancer.^8^, ^43^ Among the pathogenic variant carriers identified in our series, five of six patients were diagnosed with triple‐negative BC, and five of six patients were diagnosed with the disease before the age of 50. The combined characteristics of these patients strongly suggest a possible genetic predisposition to BC. Furthermore, two of six *BRCA1/2* pathogenic variant carriers reported a family member who died of BC (Figure 3). These two patients were from rural areas of Tanzania and had at least one family member that died of possible undiagnosed cancer, as rural area dwellers are described with limited cancer awareness and limited reach to cancer diagnostic facilities.

A very low number of VUS was observed in our analysis. We noted one VUS: the c.4042T>A (p.Cys1348Ser) in exon 11 of *BRCA2* in one patient. Our fewer VUS finding is consistent with that of the Moroccan population,^34^ and contrary to that observed in the young Rwandan population.^12^ While there is insufficient evidence to determine the role of this variant in BC, its clinical significance remains elusive. Of a special interest, the patient harboring the only VUS in our cohort was diagnosed with triple‐negative BC at 29 years of age. She was also determined to harboring the pathogenic variant: c.5123C>A (p.Ala1708Glu). We suggest a close follow‐up of this patient and her family to get more insight of the *BRCA1/2* variants in her blood relatives.

In our series, we also aimed to identify CNVs in *BRCA1/2* genes contributing to BC susceptibility. We used MLPA assay to validate the CNV results obtained after NGS analysis. We did not confirm the presence of any CNVs in either *BRCA1/2* among the 100 BC patients. The prevalence of CNVs has been investigated in the North African populations and elsewhere yielding contradicting results, most notably the absence of CNVs in *BRCA2*.^19^, ^20^ Similar to our findings, a study in Morocco reported the absence of CNVs in both *BRCA1/2* genes in patients with high risk for HBOC.^34^ In China, the CNVs prevalence of 16.1% and 0% was observed in *BRCA1* and *BRCA2*, respectively.^15^ Findings from a Turkish cohort of patients at high risk for HBOC reported a low CNVs prevalence of 2%, detected only in *BRCA1*.^44^ A study from the Republic of South Africa involving mixed races of Black Africans (277), Indians (140), White non‐Afrikaners (85), White Afrikaners (110), and colored (132) revealed the overall low prevalence of 1.1% (8/744). Two of 277 (0.7%) black Africans in their series were positive for CNVs.^22^ Taken together, our results and that from the Republic of South Africa, the prevalence of CNVs in *BRCA1/2* genes among black Africans seems to be lower. The role of *BRCA1/2* CNVs among black Africans needs further investigation in larger cohorts in order to understand their contribution to BC incidence.

Furthermore, in our cohort, we analyzed the association of some clinico‐histopathological characteristics with identified germline *BRCA1/2* pathogenic variants. We found an association between the molecular subtype and the *BRCA1/2* pathogenic variants' carrier status. Our data showed that the *BRCA1/2* pathogenic variant carriers were more likely to be diagnosed with the triple‐negative subtype of BC, which is known as an aggressive subtype with limited therapeutic options. The triple‐negative tumors lack the expressions of ER, PR, and HER‐2 markers, thus, do respond to neither hormonal therapy such as tamoxifen nor HER‐2 therapy such as trastuzumab^45^: the most attainable therapeutic options in SSA. Our results relate to those reported in Morocco,^34^ and China.^15^ Thus, this study recommends the establishment of *BRCA1/2* genetic testing as a component of BC treatment package in Tanzanian population, particularly in patients diagnosed with triple‐negative subtype.

Although our findings report an overall of 6% prevalence of germline SNV/indel pathogenic variants and CNVs in *BRCA1/2* among Tanzanian BC patients, this figure cannot adequately account for hereditary BC cases in Tanzania. It is well established that some BC patients who test negative for *BRCA1/2* pathogenic variants could be carriers of various pathogenic variants in other BC predisposition genes such as *TP53*, *PALB2*, *PTEN*, *ATM*, *STK11*, *CHEK2*, and others.^12^, ^33^, ^46^ A multi‐gene panel analysis is highly recommended to give a comprehensive picture of hereditary BC cases in Tanzania.

Our study ranks among the very few based in SSA that explore prevalence and spectrum of germline SNV/indel *BRCA1/2* pathogenic variants and CNVs among indigenous (Black) Africans. However, it comes with a few limitations. Firstly, our sample size is relatively small, involving only 100 BC patients, and this mainly was due to budget constraints. Secondly, our cohort was recruited at the only tertiary cancer‐specialized public facility (ORCI). There are a number of BC patients from regional hospitals across the country referred to this cancer‐specialized facility for chemotherapy, radiotherapy, hormonal therapy, or palliative care, but do not make it due to social and financial reasons. Therefore, our study might have missed the representation of these patients.

## 5 CONCLUSIONS

This is the first study in which both germline SNV/indel pathogenic variants and CNVs in *BRCA1/2* genes have been analyzed through NGS, Sanger sequencing, and MLPA technologies in Tanzanian women with BC. Our findings showed a relatively high overall prevalence (6%) of germline *BRCA1/2* pathogenic variants detected in six unrelated patients. We did not detect any novel germline *BRCA1/2* pathogenic variant in our cohort, therefore, the spectrum of pathogenic variants identified in this study does not differ from others in the literature. Our findings support the importance of genetic risk factors in the etiology of BC in Tanzanian population and furthermore, suggest the absolute necessity of improving genetic cancer risk assessment such as *BRCA1/2* genetic testing and counseling services for BC patients in Tanzania. The prevalence assessment of novel and/or recurrent *BRCA1/2* pathogenic variants in the population of Tanzania will enable the development of population‐specific, inexpensive genetic tests and will enhance the use of personalized treatment in Tanzania. Thus, the move to individualized BC treatment can be realized in the near future.

## AUTHORS' CONTRIBUTIONS

LPR and FA conceived the study and design; LPR, LAN, and ND enrolled patients and collected their clinical data; LPR, BKG, HY, and FA carried out laboratory analyses; BKG, SLL, and HY performed bioinformatics analysis; LPR, GA, and FA performed statistical analysis; LPR, FA, GA, and HY wrote the first draft of the manuscript. All authors read and approved the final manuscript.

## CONFLICT OF INTEREST

The authors declare no conflicts of interest.

## ETHICAL APPROVAL STATEMENT

The study protocol was approved by the Institutional Review Board of Ocean Road Cancer Institute (10/Vol/XX/16), and National Institute for Medical Research (NIMR/HQ/R.8a/Vol.IX/3255). Written informed consent was obtained from each participant.

## Supporting information


Table S1
Figure S1

## Data Availability

The data generated and/or analyzed during the present study are available from the corresponding authors on reasonable request.
