# Supplementary material for: Breast cancer in East Africa: Prevalence and spectrum of germline SNV/indel and CNVs in BRCA1 and BRCA2 genes among breast cancer patients in Tanzania
Source: Cancer Med. 2022 Jul 31;12(3):3395–409. doi: 10.1002/cam4.5091 (PMC9939169; doi:10.1002/cam4.5091)
Supplement: Supplementary file 1 — Table S1 Figure S1 [file CAM4-12-3395-s001.docx]

**Supplementary Table 1:** Primers for Sanger Sequencing to confirm NGS-detected *BRCA1/2* pathogenic variants

| Primer name | Sequence 5’---3’ | Target pathogenic variant |
| --- | --- | --- |
| BRCA1_rs80358042_Primer F | AGGGCCTTCACAGTGTCCTT | c.212+1G>A |
| BRCA1_rs80358042_Primer R | CCTACTGTGGTTGCTTCCAACC |  |
| BRCA1_rs80357508_Primer F | CAGTCTGAAAGCCAGGGAGT | c.4065_4068del |
| BRCA1_rs80357508_Primer R | GGGGCAAACACAAAAACCTGG |  |
| BRCA1_rs886039996_Primer F | ACGAAAGCTGAACCTATAAGC | c.2090del |
| BRCA1_rs886039996_Primer R | GGCAAGCCTCCCCAACTTAA |  |
| BRCA1_rs80357580_Primer F | CTGAGCTGTGTGCTAGAGGT | c.5030_5033del |
| BRCA1_rs80357580_Primer R | GCAGCAGATGCAAGGTATTCTG |  |
| BRCA1_rs28897696_Primer F | GCTGAGTTTGTGTGTGAACGG | c.5123C>A |
| BRCA1_rs28897696_Primer R | GGTGTTAAAGGGAGGAGGGGA |  |
| BRCA2_rs80359605_Primer F | TGGGAAAAGAACAGGCTTCACC | c.6591_6592del |
| BRCA2_rs80359605_Primer R | GAATGTGTGGCATGACTTGGC |  |


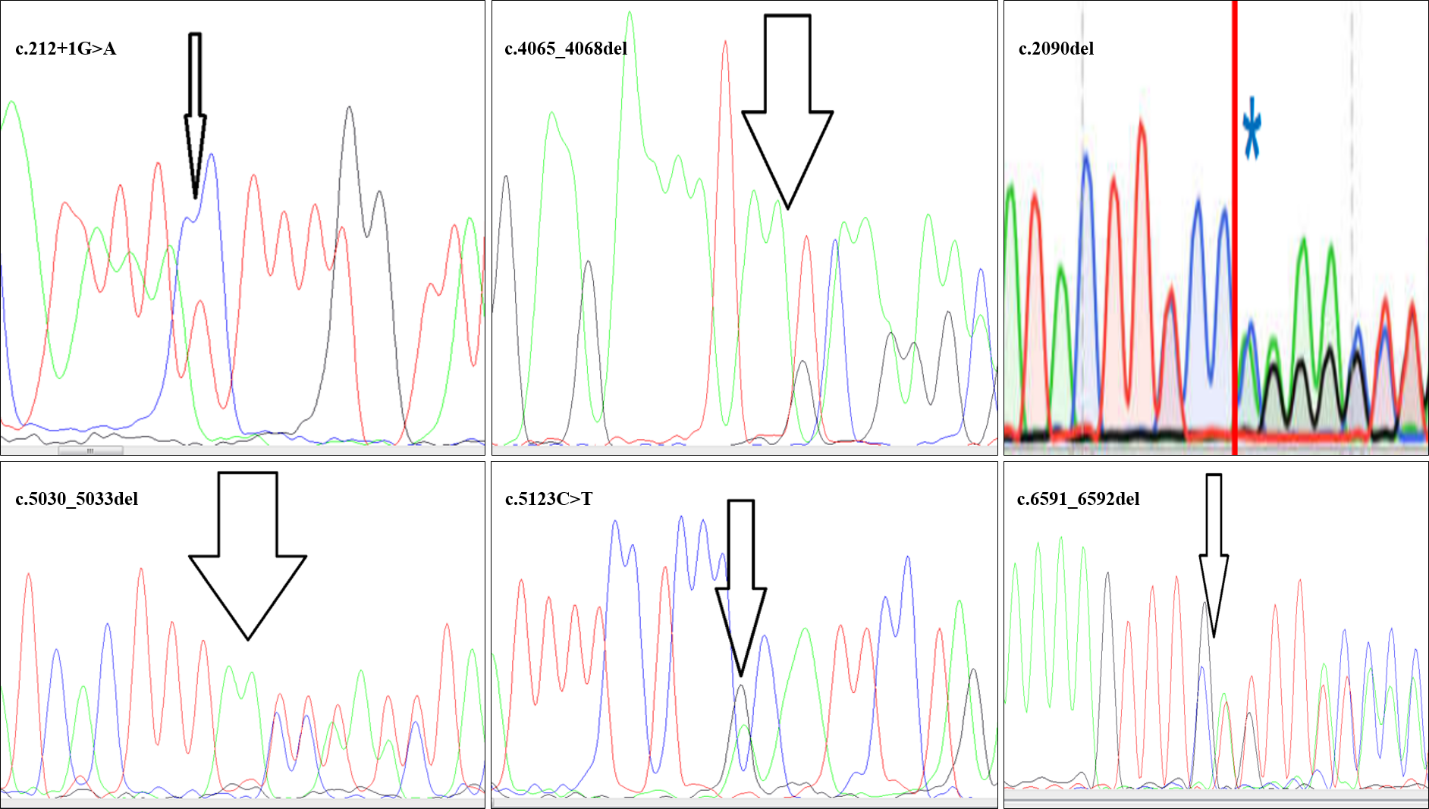


**Supplementary Figure 1:** Sanger sequencing chromatograms of *BRCA1/2* pathogenic variants detected among 100 breast cancer patients in Tanzania. An arrow or asterisk shows the position where the variation occurs.
